# RETRACTION: Wound Infection in Robotic‐Assisted Radical Prostatectomy Compared with Retropubic Radical Prostate Surgery: A Meta‐Analysis

**DOI:** 10.1111/iwj.70190

**Published:** 2025-01-12

## Abstract

**RETRACTION**: ZhuW. , WuL. , XieW. , ZhangG. , GuY. , HouY. , HeY. , “Wound Infection in Robotic‐Assisted Radical Prostatectomy Compared with Retropubic Radical Prostate Surgery: A Meta‐Analysis,” International Wound Journal 20, no. 9 (2023): 3550–3557, 10.1111/iwj.14228.37675805 PMC10588328

The above article, published online on 07 September 2023, in Wiley Online Library (http://onlinelibrary.wiley.com/), has been retracted by agreement between the journal Editor in Chief, Professor Keith Harding; and John Wiley & Sons Ltd. Following an investigation by the publisher, all parties have concluded that this article was accepted solely on the basis of a compromised peer review process. The editors have therefore decided to retract the article. The authors did not respond to our notice regarding the retraction.

**RETRACTION**: W. Zhu , L. Wu , W. Xie , G. Zhang , Y. Gu , Y. Hou , Y. He , “Wound Infection in Robotic‐Assisted Radical Prostatectomy Compared with Retropubic Radical Prostate Surgery: A Meta‐Analysis,” International Wound Journal 20, no. 9 (2023): 3550–3557, 10.1111/iwj.14228.37675805 PMC10588328

The above article, published online on 07 September 2023, in Wiley Online Library (http://onlinelibrary.wiley.com/), has been retracted by agreement between the journal Editor in Chief, Professor Keith Harding; and John Wiley & Sons Ltd. Following an investigation by the publisher, all parties have concluded that this article was accepted solely on the basis of a compromised peer review process. The editors have therefore decided to retract the article. The authors did not respond to our notice regarding the retraction.

